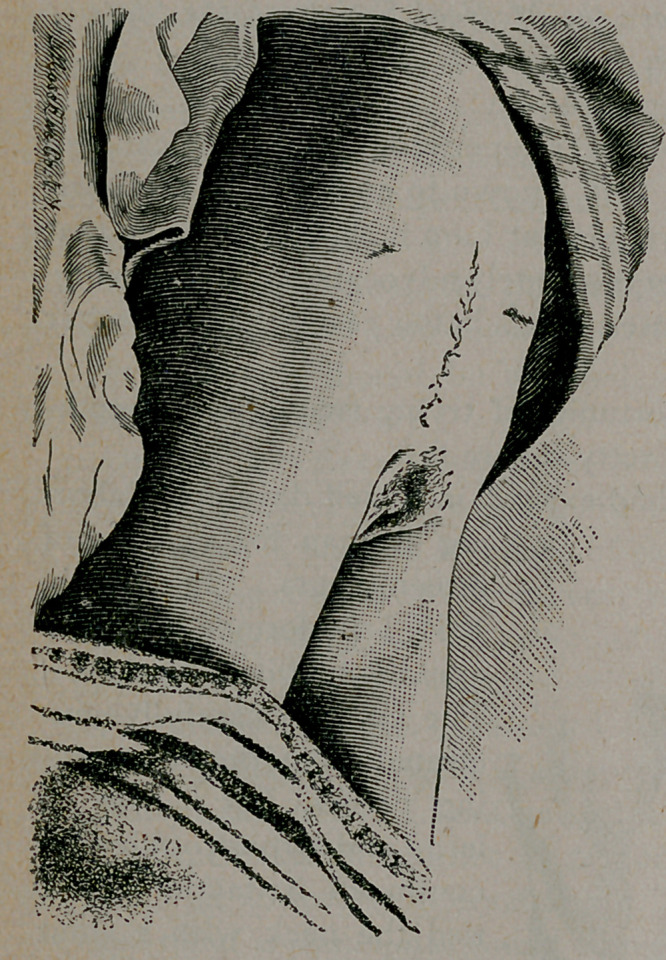# Modification of MacEwing’s Operation for the Radical Cure of Hernia

**Published:** 1887-06

**Authors:** Charles N. Jones

**Affiliations:** Surgeon to Woman’s Hospital, Brooklyn, N. Y., etc.


### MODIFICATION OF MACEWING'S OPERATION FOR THE RADICAL CURE OF HERNIA.

By CHARLES N. JONES, M. D.,
Surgeon to Woman's Hospital, Brooklyn, N. Y., etc.

*Abstract of Paper read before the Medical Society of the State of New York, February 3, 1887.*

The results following the different operations for the radical cure of hernia are not sufficiently well known to enable us to say positively which is the best operation, but I am inclined to believe that some modification of the operation lately advocated by MacEwing, of Glasgow,* will, in most cases, give a better result than any other treatment thus far known.

I have lately had the opportunity of trying two different methods, and putting them to a pretty severe test.

A large inguinal hernia had existed in a well-developed laboring woman for a period of fifteen years. In March, 1886, I performed Czerny's operation for its radical cure. The sac was carefully enucleated from the surrounding tissues, but it was so large and hypertrophied by continued irritation that I tied the neck with a stout silk ligature, using Tait's Staffordshire knot. The pillars of the inguinal canal were then united with silver wire, silk-worm and catgut suture. Patient did perfectly well, and the dressings were not removed until the eighth day, and then because the patient had stained a portion of the dressing with urine. Wound was found united throughout the entire extent. A stitch-hole abscess and sinus soon developed in the tract

* See Annals of Surgery for August, 1886.

of one of the wire sutures. Six weeks after the operation I cut down on the sinuous tract and removed the silk ligature. The softened tissues were curetted away, and the large wound packed with iodoform gauze. The patient rapidly recovered, and resumed her occupation, that of a wash-woman.

In six months a small hernia returned at the seat of the cicatrix.

In November I again operated, intending to do MacEwing's new operation, but met with unforseen difficulties. The new sac was so thin and adherent in the line of the cicatrix that, in my attempts to enucleate it, I opened into the peritoneal cavity several times. I then determined to make an elliptical section of the whole abdominal wall, through the peritonæum and including the seat of the hernial sac, performing a true laparotomy. I then united, first, the peritonæum by a continuous catgut suture.

Now follows what I believe to be the important part of MacEwing's operation, the uniting of the fibrous aponurotic structures in a valve like manner. Instead of using a needle mounted on a handle as recommended by MacEwing, I used a suture of catgut with a needle threaded at both ends. Both needles were then passed, from within, outward through the internal pillar and then through the external pillar; four sutures were thus passed, when all were tightened and tied. Poupart's ligament was found to overlap the conjoint tendon in a valve-like manner, thus securing a broad surface for union. This seems to me a more simple operation than that recommended by MacEwing. The superficial structures were then united with catgut sutures and the whole seat of operation firmly matted together by a number of buried sutures after Martin's "darning" method. It is now two months since the first operation, and there is no sign of a return of the hernial protrusion.

I would suggest, in this connection, that if MacEwing's method of uniting the tendinous pillars were adopted in ordinary laparotomies, ventral hernia would be less common. It has always been my intention to securely coapt the fibrous tissues, and in six laparotomies I have had no sign of a ventral hernia in either case.

The photographs show the original condition of the hernia, and the result two months after the final operation.

The following case illustrates the method of procedure in a case of umbilical hernia ; operation for the radical cure of irreducible umbilical hernia ; removal of sac ; suture of peritoneum and tendinous tissues ; cure :

L—— S——, married ; thirty-nine years of age ; no children. Umbilical hernia appeared eight years ago ; during the last six weeks had become inflamed and irreducible. Operation, vertical incision down to the sac ; separation of tissues from margin of tendinous ring ; sac opened ; adherent omentum separated, ligatured and excised ; margin of peritoneum sutured at level of abdominal parieties with a continuous catgut suture ; tendinous fascia united according to MacEwing's method ; other tissues united layer by layer with continuous catgut sutures ; three rubber drains inserted; antiseptic dressing applied ; minute antiseptic details followed throughout the operation. Dressing removed on the eighth day ; primary union ; bowels moved naturally on the fourth day. Recovery without incident.

The radical operation is justifiable in the following conditions :

1. Irreducible herniæ.
2. Congenital herniæ.
3. Large herniæ which cannot be restrained by the use of a truss.
4. All painful herniæ.
5. Herniæ existing in persons whose occupation subjects them to the dangers of a strangulated hernia.

CONCLUSION :

1. The operation should be performed under the most rigid and minute antiseptic precautions.
2. It is to all intents and purposes an abdominal section, and should preferably be performed by those who have been successful in this class of operations.

Under these conditions the operation becomes a very safe one.

## Figures and Tables

**Figure f1:**
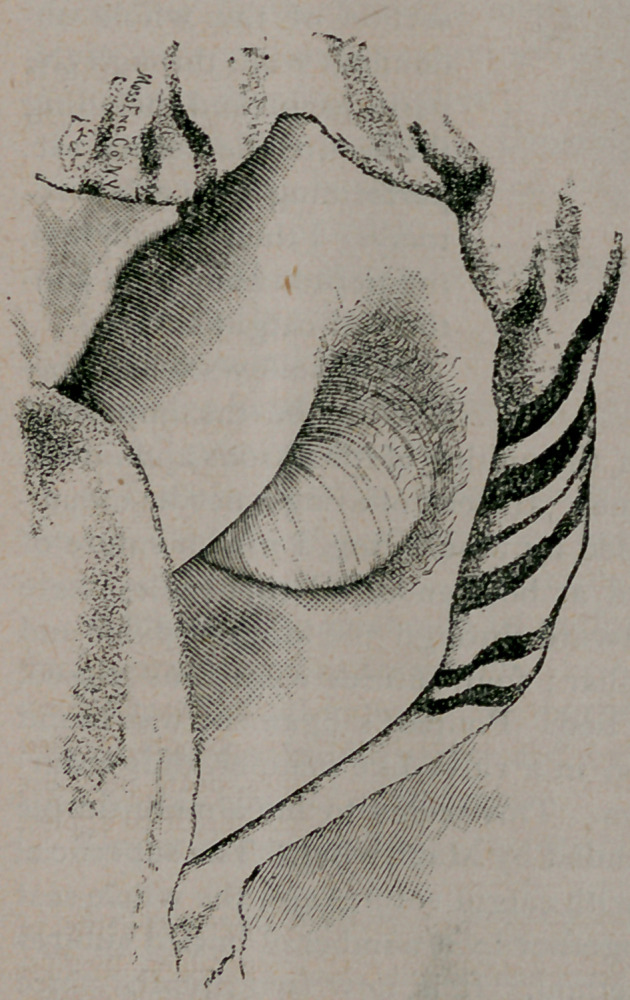


**Figure f2:**